# Synergistic effect and resistance prevention activity of antimicrobial peptide MPX with Tulathromycin against *Actinobacillus pleuropneumoniae*

**DOI:** 10.3389/fmicb.2026.1802629

**Published:** 2026-04-29

**Authors:** Hongjuan Wang, Lingcai Wang, Wei Zhang, Baoxia Chen, Rongxia Guo, Longfei Zhang, Yongli Hua

**Affiliations:** 1 Institute of Traditional Chinese Veterinary Medicine, College of Veterinary Medicine, Gansu Agricultural University, Lanzhou, China; 2 College of Animal Science and Veterinary Medicine, Henan Institute of Science and Technology, Xinxiang, China; 3 Henan International Joint Laboratory of Animal Health Breeding and Disease Prevention and Control, Xinxiang, China; 4 Lanzhou Veterinary Research Institute, Chinese Academy of Agricultural Sciences (LVRI, CAAS), Lanzhou, China

**Keywords:** *Actinobacillus pleuropneumoniae*, antimicrobial peptide MPX, resistance prevention, synergy effect, Tulathromycin

## Abstract

**Introduction:**

The emergence and dissemination of multidrug-resistant *Actinobacillus pleuropneumoniae* (APP) have significantly hindered the advancement of the swine industry. The combination of antimicrobial peptides (AMPs) with conventional antibiotics represents a promising strategy to combat drug-resistant bacterial infections. Tulathromycin (Tul), however, is prone to inducing resistance in APP due to its broad mutant selection window (MSW). Moreover, there is a paucity of data regarding the efficacy of AMP-macrolide combinations against APP. Consequently, this study aimed to evaluate the synergistic effects and resistance prevention of the AMP MPX in combination with Tul against APP.

**Methods:**

To assess synergism, the minimum inhibitory concentrations (MICs) of MPX and Tul, both individually and in combination, were determined using the micro-broth dilution method and checkerboard assay. Time-kill assays were conducted to analyze antibacterial activity and kill rate by quantifying viable bacterial counts. The post-antibiotic effect (PAE) was calculated following exposure to 1−2 MIC of each drug alone and in combination. For resistance prevention analysis, serial passage experiments were performed over 30 generations under selective pressure at 1/4 MIC for each agent to monitor changes in MIC values. Additionally, the mutant prevention concentration (MPC) was measured to evaluate MPX’s capacity to narrow the MSW of Tul against APP. Finally, the antimicrobial activity of MPX and Tul was assessed against resistant APP strains.

**Results and discussion:**

The fractional inhibitory concentration index (FICI) indicated an indifferent interaction (1.5) for susceptible strains and an additive effect (0.75) for resistant strains. Time-kill curves demonstrated that MPX significantly enhanced the antibacterial efficacy of Tul against APP. Specifically, the kill rate within 0−1 hour and the PAE at 1−2 MIC of Tul increased from 0.35−2.91 to 1.41−5.03 Log_10_ CFU/mL/h and from 0.66−1.64 to 1.29−2.91 h, respectively, combination with MPX. Resistance induction assays revealed that MPX could restore or reduce the MIC of Tul against APP. Furthermore, MPX effectively narrowed the MSW of Tul, as evidenced by an MPC-based FICI of 0.375, confirming a synergistic interaction. The combination exhibited additive effects against resistant strains. These findings provide valuable insights into the potential application of MPX in combination with antimicrobial agents to prevent the emergence and dissemination of drug-resistant strains.

## Introduction

Porcine contagious pleuropneumonia (PCP) represents a critical respiratory illness in swine, attributable to the pathogen *Actinobacillus pleuropneumoniae* (APP) (To et al., 2025; Stringer et al., 2022). Vaccination constitutes an effective prophylactic strategy. Nevertheless, the extensive diversity of APP serotypes and the limited cross-protective efficacy among them necessitate the continued reliance on antimicrobial drugs (AMDs) for treatment (Scherrer et al., 2022). Commonly employed AMDs include cephalosporins, fluoroquinolones, and macrolides. Notably, inappropriate or non-standardized administration of these agents has contributed to the emergence and spread of multidrug-resistant (MDR) APP strains (Hennig-Pauka et al., 2022; Guarneri et al., 2024; Paulina and Dawid, 2025; Vilaró et al., 2024). In Poland, the resistance rates of isolated APP were reported as 55.5%, 36.1%, 32.8%, and 26.1% for tylosin, gentamicin, doxycycline, and sulfamethoxazole/trimethoprim, respectively, and the prevalence of multidrug resistance fluctuated between 14.3% and 21.9% during the period from 2019 to 2024 (Paulina and Dawid, 2025). In Italy, the most frequently observed resistances were against tetracycline (53%) and ampicillin (33%), followed by enrofoxacin, forfenicol, trimethoprim/sulfamethoxazole (23% each), and multidrug resistance was also prevalent with approximately more than three classes of AMD between 2015 and 2022 (Guarneri et al., 2024). This resistance significantly limits the available clinical interventions for managing MDR infections, often leading to treatment failure and representing a critical risk to both food safety and public health. Consequently, addressing the challenge of diminishing effective AMDs due to bacterial resistance has become an urgent priority.

Antimicrobial peptides (AMPs) is a key antibiotic alternatives or antibiotic adjuvants to address multidrug resistance, capable of inhibiting biofilm formation and disrupting microbial membranes. These actions effectively counteracting resistance mechanisms commonly observed in Gram-negative (G^−^) bacteria. Nonetheless, AMPs exhibit certain limitations, including vulnerability to enzymatic degradation and relatively high MIC. Consequently, the strategic combination of AMPs with traditional antibiotics, which harnesses both immunological activation and enhanced antimicrobial efficacy, is essential for overcoming MDR and prolonging the clinical utility of existing antibiotics in the 21st century (Chen et al., 2024; Oliveira et al., 2025; Roque-Borda et al., 2026). For instance, AMP K11 in combination with chloramphenicol, meropenem, rifampicin, or ceftazidime can produce obviously synergistic effect against MDR and extensively drugresistant *Klebsiella pneumoniae* (Chatupheeraphat et al., 2023). Similarly, AMP P-113 and its derivatives exhibit strong synergy with vancomycin against resistant *Enterococcus faecium*, *Staphylococcus aureus*, and *Escherichia coli* (Wu et al., 2020). Additionally, AMP SLAP-S25 can boost the efficacy of antibiotics covering all major classes, including cefepime, colistin, ofloxacin, rifampicin, tetracycline, and vancomycin, against MDR G^−^ pathogens (Song et al., 2020).

Mastoparan X (MPX), a 14-amino acid peptide belonging to the wasp venom AMP family, demonstrates significant antibacterial efficacy by disrupting bacterial cell membranes and biofilms. It exhibits potent activity against a broad spectrum of G^+^ and G^−^ bacteria (Bhattacharya et al., 2025). Prior investigations have revealed that MPX effectively inhibits pathogens such as *S. aureus*, *Salmonella enterica*, *E. coli*, *Candida albicans*, and *A. pleuropneumoniae* by compromising membrane integrity, increasing membrane permeability, altering membrane electromotive force, and reducing biofilm formation (Wang et al., 2020; Zhu et al., 2023). The mutant selection window (MSW), defined by the MIC and the mutant prevention concentration (MPC), delineates the concentration range within which selective pressure may promote the emergence of resistant bacterial mutants (Krajewska et al., 2023). Within this range, susceptible bacterial populations have the potential to develop resistance. Tulathromycin (Tul) is a veterinary-specific macrolide drug used to treat porcine respiratory diseases. Our previous studies demonstrated that APP is susceptible to resistance mutations when exposed to Tul concentrations within the mid-range of the MSW (Wang and Zhang, 2024). Consequently, the present study aims to evaluate the synergistic antibacterial effects and mutant prevention potential of MPX in combination with Tul against APP.

## Materials and methods

### Bacterial strains

The standard strains of APP (CVCC259) and *S. aureus* (ATCC29213) were obtained from the China Institute of Veterinary Drug Control. The resistant strain T32 was obtained in our previous MSW study of Tul against APP.

### Drugs and reagents

Tulathromycin (purity 98%) was obtained from Shandong Lukang Selleck Pharmaceutical Co., Ltd. AMPs (MPX, AaeAp2a, BMAP-18, A5, Piscidin, L2, and BSN-37, purity ≥95%) were synthesized by Shanghai Gil Biochemical Co., Ltd. Citric acid was procured from Tianjin Tianli Chemical Reagent Co., Ltd. Tryptic Soy Broth (TSB) and Tryptic Soy Agar (TSA) media were supplied by Guangdong Huankai Microbial Science and Technology Co., Ltd. Newborn calf serum was acquired from Shanghai Yuduo Biotechnology Co., Ltd., while nicotinamide adenine dinucleotide (NAD) was provided by Shanghai Yuanye Biotechnology Co., Ltd.

### Preparation of exponential phase bacterial suspension

TSA and TSB media for the cultivation of APP were prepared in accordance with the manufacturer’s guidelines and supplemented with 4% (v/v) newborn calf serum and 1% (v/v) NAD solution (1 mg/mL). For the exponential growth phase of APP, one to three colonies grown on TSA were inoculated into 5 mL of TSB and incubated in a shaking incubator at 37 °C, 180–200 rpm for 8 h, yielding a bacterial density of approximately 10^9^ colony-forming units per milliliter (CFU/mL), as determined by the agar plate counting method.

### MIC and Synergy determination

The MICs of AMPs and Tul against CVCC259 were determined using the micro-broth dilution method in 96-well microtiter plates (90 μL/well) in accordance with Clinical and Laboratory Standards Institute (CLSI) guidelines.

Specifically, 45 μL of blank TSB was initially dispensed into wells 1–9 of the first row. Subsequently, 45 μL of drug-containing TSB (32 μg/mL) was added to the first well and mixed thoroughly. A two-fold serial dilution was then performed by transferring 45 μL of the mixture sequentially from well 1 to 9. Following this, 45 μL of a logarithmic-phase bacterial suspension, approximately 1 × 10^6^ CFU/mL, was added to wells 1–9, resulting in a final bacterial concentration of 5 × 10^5^ CFU/mL and a final drug concentration range spanning 0.031 to 8 μg/mL. Negative controls (90 μL of sterile broth) and positive controls (90 μL of bacterial suspension) were included. The plates were incubated at 37 °C for 16 to 18 h. The MIC was defined as the lowest concentration of the drug that completely inhibited visible bacterial growth. *S. aureus* ATCC 29213 served as the quality control strain.

The MICs of combinations of AMPs with Tul against CVCC259 were assessed using a checkerboard assay. Both Tul and each AMP were serially diluted two-fold in TSB to achieve the desired concentrations. Subsequently, 22.5 μL of each AMP and Tul dilution was added to sterile 96-well plates. AMP concentrations ranged from 2 to 1/64 of their respective MICs across rows 1 to 8, while Tul concentrations ranged similarly across columns 1 to 8. After mixing, 45 μL of bacterial suspension was added to each well to attain a final bacterial concentration of 5 × 10^5^ CFU/mL. The plates were incubated at 37 °C for 16 to 18 h, with negative and positive controls included in parallel.

All experiments were conducted in triplicate, and data were considered valid only when all three replicates yielded consistent results. The interaction between AMPs and Tul was quantified using the fractional inhibitory concentration index (FICI), calculated as follows: FICI = (MIC of drug A in combination / MIC of drug A alone) + (MIC of drug B in combination / MIC of drug B alone). The nature of the interaction was classified as synergistic (FICI ≤ 0.5), additive (0.5 < FICI ≤ 1), indifferent (1 < FICI ≤ 4), or antagonistic (FICI > 4).

### *In vitro* static time-kill curves

Three distinct drug groups were delineated for the study. In the Tul and MPX monotherapy groups, 1 mL of drug-containing TSB was added to a centrifuge tube with 8.9 mL of TSB, resulting in final drug concentrations ranging from 0.5 to 8 times the MIC. For the combination therapy group, 1 mL of each drug was introduced into a tube containing 7.9 mL of TSB, achieving the same final concentration range based on the combined MIC values. Corresponding antibiotic-free controls were prepared.

Subsequently, 100 μL of a bacterial suspension in the logarithmic growth phase (10^9^ CFU/mL) was added to each tube and mixed thoroughly. The tubes were incubated at 37 °C, and samples were collected at 0, 1, 3, 6, 9, and 12 h to quantify the bacterial population by plating serial ten-fold dilutions onto TSA. The detection limit was established at 50 CFU/mL, bacterial counts below this threshold were considered undetectable and were plotted as 50 CFU/mL in the time-kill curve analyses. Each experiment was conducted in triplicate. The mean bacterial population, expressed as Log_10_ CFU/mL, was plotted on the y-axis against incubation time on the x-axis to generate time-kill curves (KCs).

The antibacterial effect (E) and kill rate were subsequently calculated. The E was defined as the maximal change in bacterial count relative to the initial inoculum during 0 to 12 h. This antibacterial effect was categorized as bacteriostatic (0 Log_10_ CFU/mL reduction), bactericidal (−3 Log_10_ CFU/mL reduction), and eradication (−4 Log_10_ CFU/mL reduction). The kill rate was defined as the difference between the initial and final bacterial counts within a specified time interval, divided by the duration of that interval.

### Post antibiotic effect determination

For Tul and MPX alone group: 500 μL of Tul or MPX was added to a centrifuge tube containing 4 mL of TSB to yield final concentrations of 1 and 2 MIC. For combination group: Based on the combined MIC, 500 μL of each drug was added to a centrifuge tube containing 3.5 mL of TSB broth to yield final concentrations of 1 and 2 MIC. Control groups without antibiotic exposure were included.

Subsequently, 500 μL of a logarithmic-phase bacterial suspension (10^8^ CFU/mL) was introduced into each tube, followed by static incubation at 37 °C for 2 h. After incubation, 100 μL of the bacterial suspension was transferred into 10 mL of fresh TSB to achieve a 1:1,000 dilution. The diluted cultures were then statically incubated at 37 °C, and bacterial populations were quantified at 0, 1, 2, 4, 6, and 8 h by agar plate counting method. Bacterial regrowth curves were generated based on these data.

The post-antibiotic effect (PAE) for each individual agent and their combination was calculated using the equation:


PAE=T−C

where T denotes the time required for the bacterial population (Log_10_ CFU/mL) in the drug-treated group to increase tenfold, and C represents the corresponding time for the control group.

### Resistance induction by serial passages

The capacity of MPX to resist induction against APP was evaluated through serial passaging at sub-inhibitory drug concentrations. Specifically, CVCC259 underwent 30 successive generations of cultivation in the presence of 1/4 MIC of Tul, 1/4 MIC of MPX, and a combination of 1/4 MIC Tul and MPX. In details, 0.5 mL of bacterial suspension in the logarithmic phase (approximately 10^8^ CFU/mL) was inoculated into 4.5 mL of drug-containing broth and incubated for 12 h at 37 °C, 200 rpm, resulting in the first generation. Subsequently, the same method was applied to cultivate the bacteria from the first generation up to the 30th generation. The MIC of Tul was measured every two generations. The ratio of the MIC after passage to the first generation was calculated and a graph depicting the MIC evolution was generated.

### Mutant selection window

To evaluate the anti-mutant efficacy of MPX, its capacity to close the MSW of Tul against CVCC259 was investigated. Initially, drug-containing TSA were prepared, with concentrations of Tul and MPX ranging from 0.5 to 128 times the MIC of each agent in TSB. For combination assays, the concentration of Tul was increased two-fold from 0.5 to 64 MIC, while MPX was tested between 1 and 8 MIC.

Subsequently, bacterial cultures were established by inoculating two 50 mL TSB tubes with 5–10 colonies, followed by incubation at 37 °C, 180–200 rpm for 8 h. The cultures were then centrifuged at 5000 × *g* for 20 min at 4 °C. After discarding the supernatant, the bacterial pellet was resuspended in 0.5 mL of sterile TSB to achieve a suspension with an approximate concentration of 1.5 × 10^11^ CFU/mL. 100 μL suspension was evenly spread onto each drug-containing TSA and incubated at 37 °C for 72 h, with observations recorded at 24 h intervals. The minimum concentration that completely inhibited bacterial growth was designated as the MPC. All experiments were conducted in triplicate.

To assess the propensity of the drugs to select for resistance mutations, the selection index (SI) was calculated as the ratio of MPC to MIC (SI = MPC/MIC). A higher SI value indicates an increased likelihood of resistance mutation emergence.

### Antibacterial activity against resistant APP

The antibacterial efficacy of MPX and Tul against resistant APP was evaluated utilizing the resistant strain T32, isolated in our prior MSW study. MIC, static time-kill assays, antibacterial activity and bactericidal rates were determined following the methodology for standard bacteria CVCC259.

### Data analysis

All experiments were conducted in triplicate. Data are expressed as mean ± standard deviation (SD). Statistical analyses and graphical representations were carried out using GraphPad Prism version 9.5.1. Differences between groups were evaluated using one-way analysis of variance (ANOVA) followed by Tukey’s multiple comparison test. Statistical significance was denoted as follows: * indicates a statistically significant difference (*p* < 0.05), ** indicates a highly significant difference (*p* < 0.01), and *** indicates an extremely significant difference (*p* < 0.001).

## Results

### Antimicrobial susceptibilities results

The MIC of Tul against CVCC259 was 1 μg/mL, while the MICs for the AMPs were presented in Table 1. Among these AMPs, MPX demonstrated the strongest antibacterial efficacy, with an MIC of 16 μg/mL. Consequently, MPX was chosen for subsequent studies involving combination therapy.

**Table 1:** MICs of AMPs against CVCC259 (μg/mL).

AMPs	Amino acid sequence	MIC
MPX	INWKGIAAMAKKLL	16
AaeAp2a	FLFKLIPKAIKGLVKAIRK	32
BMAP-18	GRFKRFRKKFKKLFKKIS	128
A5	RGLPODCERRGGFCSHKSCPPGIGRIGLCSKEDFCCRSRWYSDDDDK	>256
Piscidin	FFHHIFRGIVHVGKTIHRLVTG	>256
L2	MTPFWRGVSLRPVGASCRDNSECITMLCRKNRCFLRTASEDDDDK	>256
BSN-37	FRPPIRRPPIRPPFYPPFRPPIRPPIFPPIRPPFRPP	>256

The MICs and FICI values for MPX and Tul against CVCC259 and T32 are presented in Table 2. In the case of CVCC259, the combination resulted in a twofold reduction in the MIC of Tul, while the MIC of MPX remained unchanged. The calculated FICI of 1.5 suggests an indifferent interaction. Tor the T32 strain, the MIC of Tul decreased fourfold and the MIC of MPX decreased twofold when used in combination, producing an FICI of 0.75, indicative of an additive effect. Notably, MPX exhibited identical MIC values against both the susceptible and resistant strains following combination.

**Table 2:** MICs of MPX and Tul alone and combination against CVCC259 and T32.

Strains	Drugs			FICI
	Tul	MPX	Tul+MPX	
CVCC259	1	16	0.5 + 16	1.5
T32	32	32	8 + 16	0.75

### Time-kill curves of Tul and MPX against CVCC259

**Figure 1:**
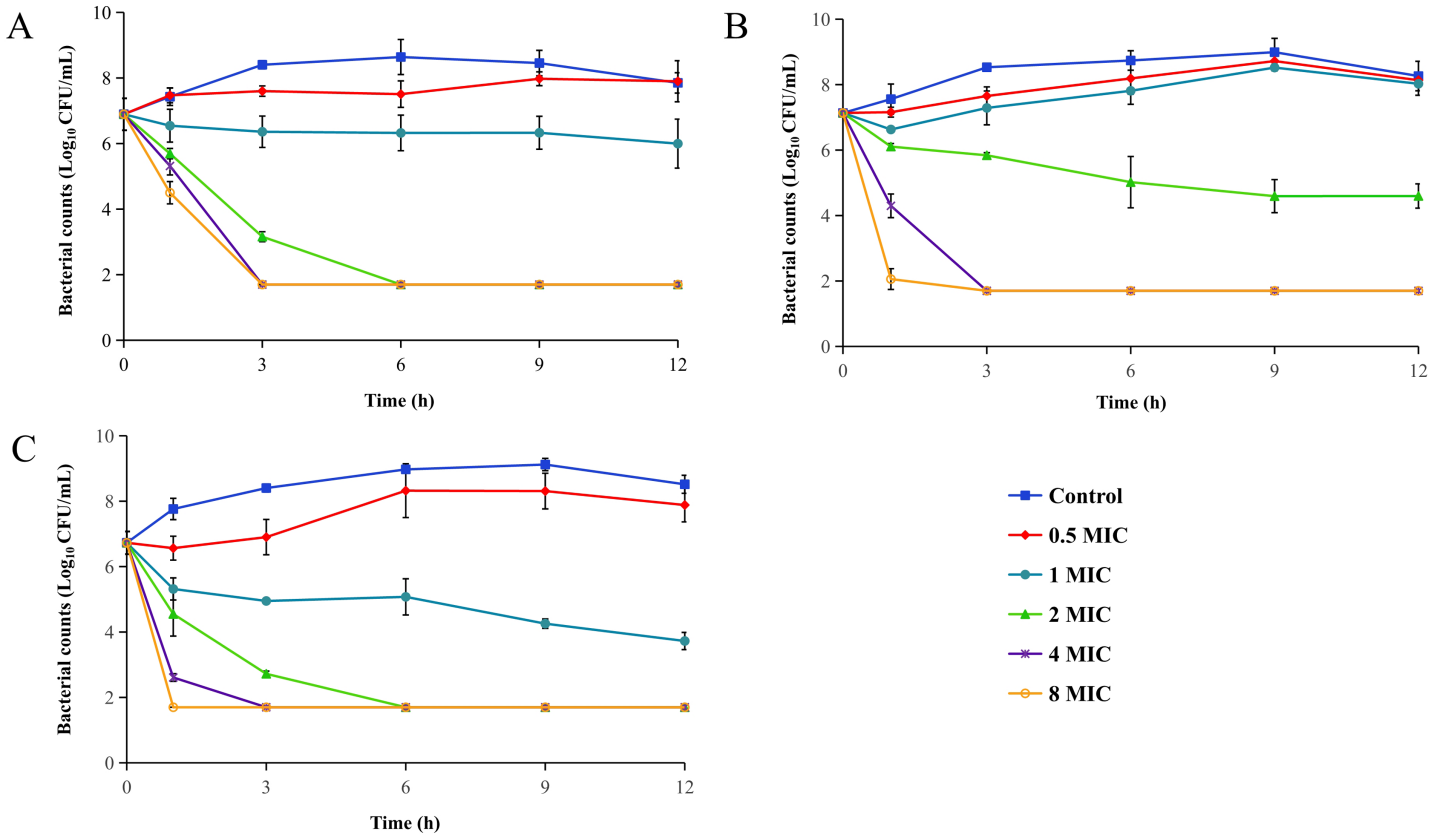
Time-kill curves of Tul and MPX alone and combination against CVCC259. **(A)** Tul alone group. **(B)** MPX alone group. **(C)** Tul combined to MPX group. All data are expressed as mean ± SD determined from three independent experiments.

The static time-kill curves of Tul, MPX, and their combination against CVCC259 were depicted in Figures 1A–C, respectively.

In Figure 1A, a bacteriostatic effect (−0.66 Log₁₀ CFU/mL) was observed at 1 MIC, while an elimination effect (−5.20 Log₁₀ CFU/mL) was achieved within 6 h at 2 MIC. Eradication occurred within 3 h at 4–8 MIC. Figure 1B shows that bacterial growth was moderately inhibited but regrew after 1 h at 0.5–1 MIC. A bacteriostatic effect (−2.54 Log₁₀ CFU/mL) was seen at 2 MIC, and a bactericidal effect was achieved at 4–8 MIC. Figure 1C indicates that bacterial growth was mildly inhibited but resumed after 1 h at 0.5 MIC. A bactericidal effect (−3.0 Log₁₀ CFU/mL) was achieved at 1 MIC, and bactericidal activity was attained at 2–8 MIC.

The E and kill rate data were summarized in Table 3. Table 3 shown that MPX can significantly enhanced the E of Tul against CVCC259 at 1 MIC. When exceed 2 MIC, no differences were observed between Tul alone and combination.

**Table 3:** E (Log_10_ CFU/mL) and kill rate (Log_10_ CFU/mL/h) during 0–1 h of Tul and MPX alone and combination against CVCC259.

PD indices	Drugs	Concentrations					
		0	0.5 MIC	1 MIC	2 MIC	4 MIC	8 MIC
E	Tul	2.04 ± 0.46	1.21 ± 0.22	−0.66 ± 0.27^***^	−5.20 ± 0.49	−5.20 ± 0.49	−5.20 ± 0.49
	MPX	1.86 ± 0.45	1.59 ± 0.18	−0.50 ± 0.11^***^	−2.54 ± 0.36^***^	−5.44 ± 0.03	−5.44 ± 0.03
	Tul + MPX	2.39 ± 0.45	1.59 ± 0.80	−3.00 ± 0.24	−5.03 ± 0.35	−5.03 ± 0.35	−5.03 ± 0.35
Kill rate	Tul	0.53 ± 0.25	0.58 ± 0.04	−0.35 ± 0.05 ^*^	−1.20 ± 0.15^*^	−1.58 ± 0.21 ^***^	−2.39 ± 0.82^***^
	MPX	0.42 ± 0.49	0.02 ± 0.13	−0.50 ± 0.11 ^*^	−1.03 ± 0.12 ^**^	−2.84 ± 0.33 ^**^	−5.08 ± 0.35
	Tul + MPX	1.03 ± 0.43	−0.16 ± 0.34	−1.41 ± 0.09	−2.18 ± 0.20	−4.12 ± 0.24	−5.03 ± 0.35

All experiments were conducted in triplicate. Data are expressed as mean ± SD. *indicates a statistically significant difference (*p* < 0.05), **indicates a highly significant difference (*p* < 0.01), and ***indicates an extremely significant difference (*p* < 0.001).

Given that the bacterial population declined below the detection threshold after 3 h at elevated drug concentrations, the kill rate during the initial 0–1 h interval was calculated to more precisely characterize the antibacterial properties of each treatment. The findings indicate that the kill rates of Tul and MPX, both individually and in combination, progressively increased with rising drug concentrations, demonstrating concentration-dependent activity. At equivalent concentrations, the kill rate observed for the combination treatment was significantly greater than that of either agent administered alone.

Importantly, although the FICI suggested an indifferent interaction, the time-kill curve assay conducted at 1 MIC and the kill rate within the 0–1 h interval indicated a significantly enhanced effect. This discrepancy suggests that exclusive reliance on the FICI for assessing combination efficacy may lead to biased conclusions.

### Post antibiotic effect

**Figure 2:**
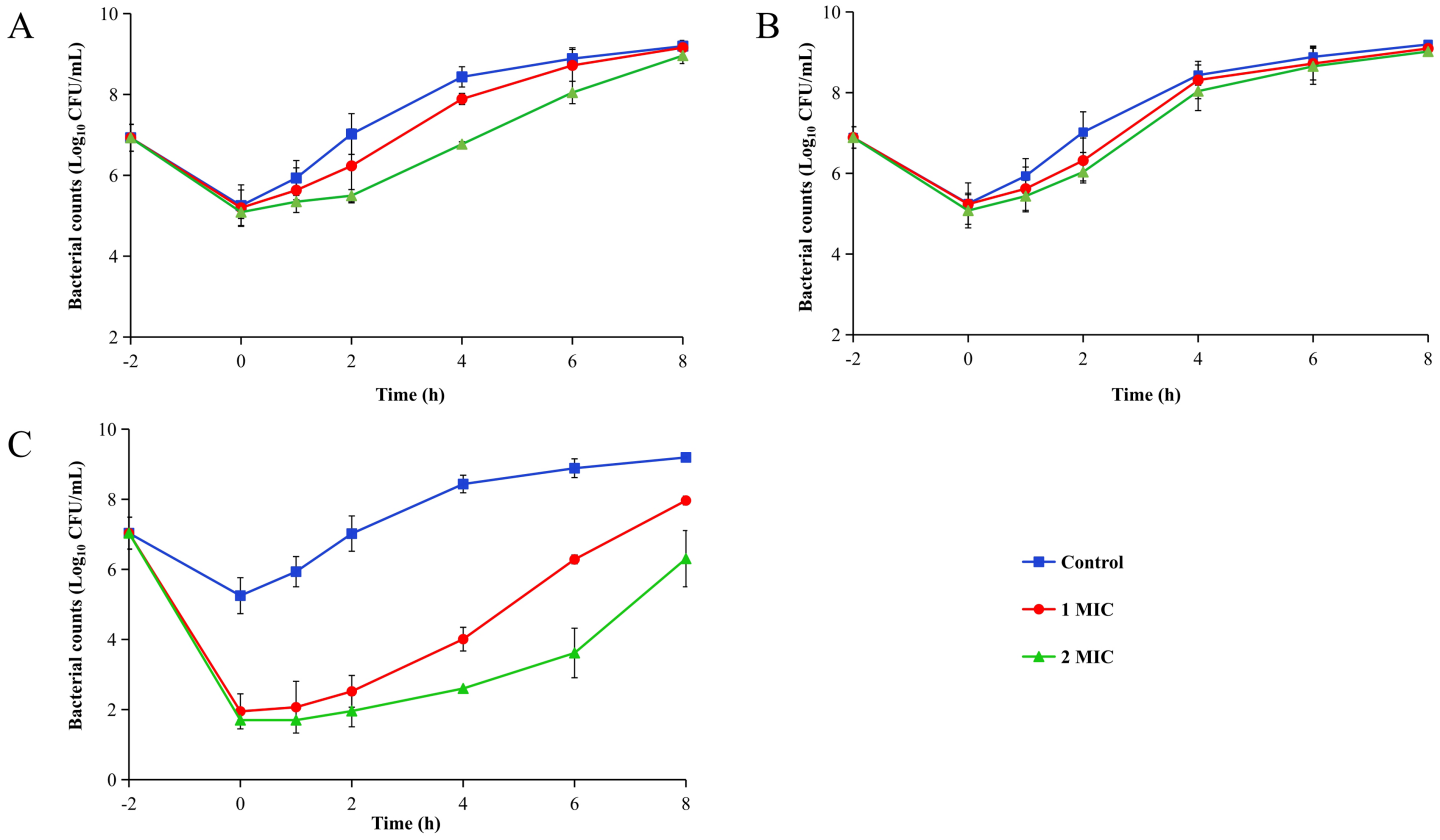
Post antibiotic regrowth curves of MPX and Tul alone and combination against CVCC259. **(A)** Tul alone group. **(B)** MPX alone group. **(C)** Tul combined to MPX group. All data are expressed as mean ± SD determined from three independent experiments.

The regrowth curves and PAE values were shown in Figure 2 and Table 4, respectively. Tul alone (Figure 2A) and the combination (Figure 2C) at 2 MIC exhibited markedly slower recovery than at 1 MIC. In contrast, the MPX alone group (Figure 2B) showed little difference between the two concentrations.

**Table 4:** PAE (h) of MPX and Tul alone and combination against CVCC259.

Drugs	Concentrations	
	1 MIC	2 MIC
Tul	0.66 ± 0.17^*^	1.64 ± 0.18^**^
MPX	0.60 ± 0.37^*^	0.76 ± 0.29^***^
MPX + Tul	1.29 ± 0.10	2.91 ± 0.35

Experiment were conducted in triplicate. Data are expressed as mean ± SD. *indicates a statistically significant difference (*p* < 0.05), **indicates a highly significant difference (*p* < 0.01), and ***indicates an extremely significant difference (*p* < 0.001).

The PAE values were listed in Table 4. Table 4 shows that for the individual agents, the PAE of Tul at 2 MIC was significantly longer than at 1 MIC (1.64 h vs. 0.65 h), whereas MPX showed minimal variation (0.76 h vs. 0.60 h). The combination of Tul with MPX significantly prolonged the PAE of Tul.

### Resistance induction by serial passages

**Figure 3:**
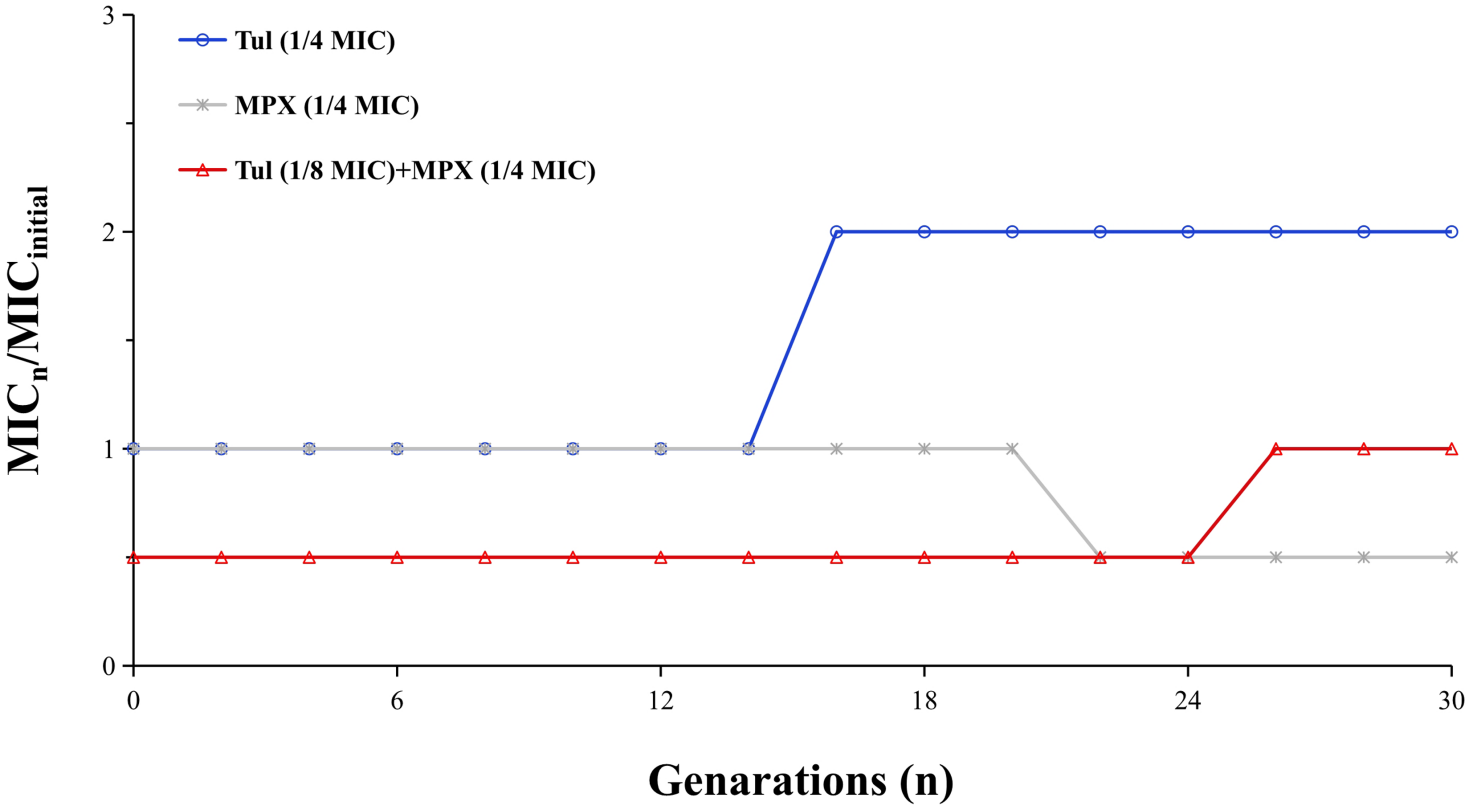
The MIC changes of APP induced by MPX and TUL alone and combination after 30 consecutive generations of treatment. The change in MIC was described by normalizing the MIC of *n* generation to the MIC of first generation.

Figure 3 illustrates the changes in the MIC of Tul against APP over 30 serial passages at sub-inhibitory concentrations. When administered alone, Tul demonstrated a two-fold increase in MIC after the 14th passage. Conversely, the MIC of MPX alone decreased two-fold by the 22nd passage. In the combination treatment group, the MIC decreased two-fold up to the 24th passage, subsequently returning to the initial baseline level. These results suggest that the combination of MPX with Tul may partially retard the development of antimicrobial resistance.

### Mutant selection window

The MPCs and SI were listed in Table 5. The MPC of Tul and MPX alone against CVCC259 were 64 and 512 μg/mL, respectively. In combination, the MIC and MPC were 0.5 and 16 μg/mL for Tul, 16 and 64 μg/mL for MPX, respectively. Consequently, the MSW for Tul narrowed substantially from 1–64 μg/mL to 0.5–16 μg/mL. The MPC based FICI was 0.375, indicating a synergy effect.

**Table 5:** MSW of MPX and Tul alone and combination against CVCC259.

Drugs	MSW parameters		
	MIC (μg/m)	MPC (μg/m)	SI
Tul	1	64	64
MPX	16	512	32
Tul + MPX	0.5 + 16	16 + 64	32 + 4

### Antibacterial activity of MPX and Tul against T32

**Figure 4:**
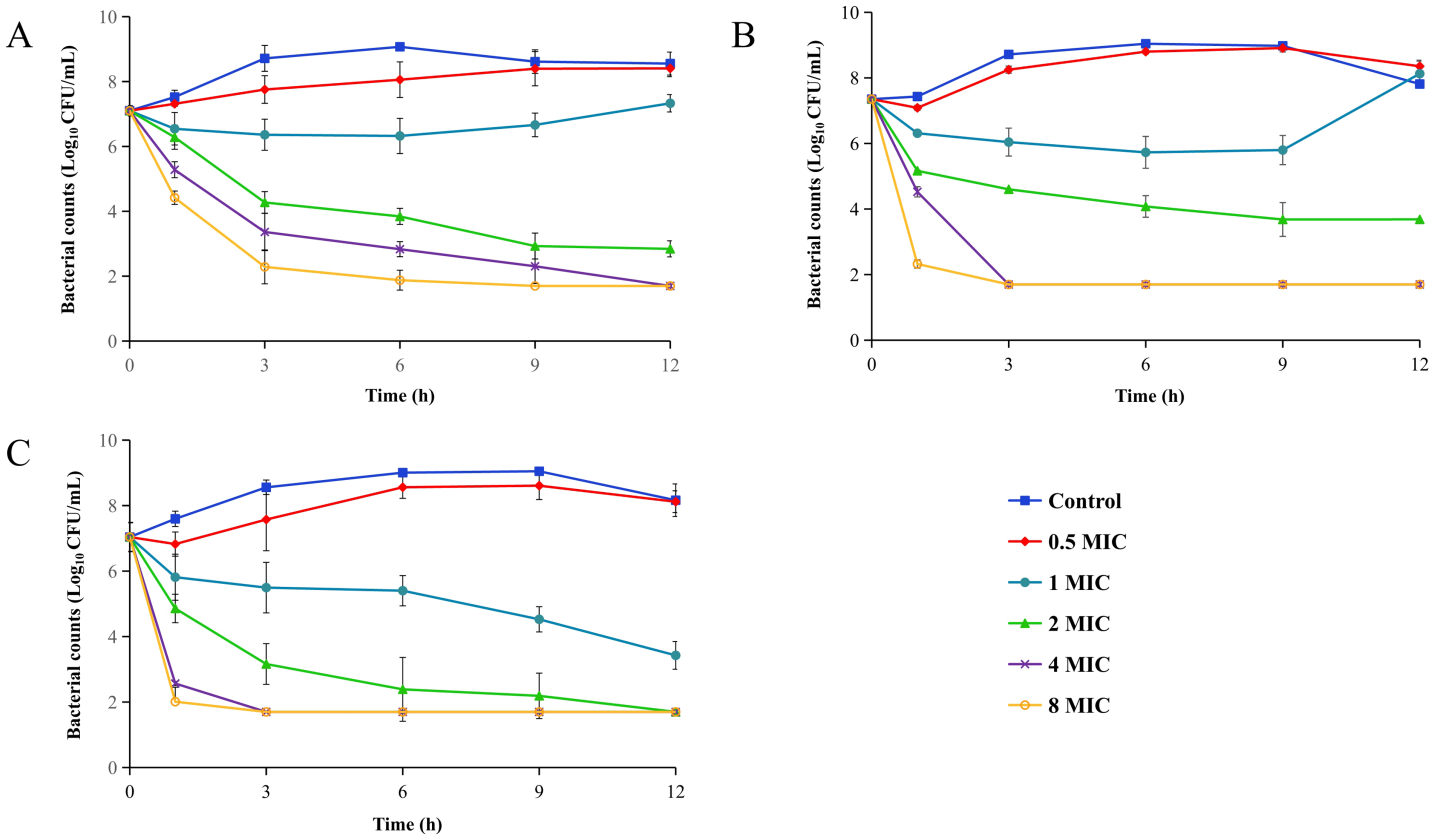
Time kill curves of Tul and MPX alone and combination against T32. **(A)** Tul alone group. **(B)** MPX alone group. **(C)** Tul combined to MPX group. All data are expressed as mean ± SD determined from three independent experiments.

The time-kill curves of MPX and Tul alone and in combination against T32 were presented in Figure 4. The E and kill rate during 0–1 h were listed in Table 3.

Figure 4A illustrates that the antibacterial activity of Tul increased proportionally with its concentration. Bacterial regrowth was observed after 3 h at 1 MIC, whereas an eradication effect (> − 4.26 Log₁₀ CFU/mL) were achieved when the concentrations exceeding 2 MIC. Similarly, MPX (Figure 4B) demonstrated a concentration-dependent antibacterial effect against strain T32, with a more rapid bactericidal action compared to Tul. The combination group (Figure 4C) exhibited a markedly enhanced antibacterial effect, achieving bactericidal activity at 1 MIC (−3.61 Log₁₀ CFU/mL). The kill rate was significantly higher than that of Tul alone group (see Table 6).

**Table 6:** E (Log_10_ CFU/mL) and kill rate during 0–1 h (Log_10_ CFU/mL/h) of Tul and MPX alone and combination against T32.

PD indices	Drugs	Concentrations					
		0	0.5 MIC	1 MIC	2 MIC	4 MIC	8 MIC
E	Tul	1.97 ± 0.15	1.31 ± 0.37^***^	0.23 ± 0.10^***^	−4.26 ± 0.28^*^	−5.40 ± 0.17	−5.40 ± 0.17
	MPX	1.69 ± 0.00	−0.27 ± 0.08	−1.88 ± 0.38^***^	−3.27 ± 0.33^***^	−5.65 ± 0.00	−5.65 ± 0.00
	Tul + MPX	2.01 ± 0.54	−0.21 ± 0.07	−3.61 ± 0.86	−5.34 ± 0.44	−5.34 ± 0.44	−5.34 ± 0.44
Kill rate	Tul	0.42 ± 0.38	0.21 ± 0.05	−0.56 ± 0.14^***^	−0.82 ± 0.19^***^	−1.82 ± 0.26^***^	−2.68 ± 0.27^***^
	MPX	0.08 ± 0.00	−0.27 ± 0.08	−1.04 ± 0.06	−2.19 ± 0.02	−2.83 ± 0.16^***^	−5.03 ± 0.13
	Tul + MPX	0.56 ± 0.67	−0.21 ± 0.07	−1.23 ± 0.26	−2.18 ± 0.01	−4.47 ± 0.50	−5.03 ± 0.00

All experiments were conducted in triplicate. Data are expressed as mean ± SD. *indicates a statistically significant difference (*p* < 0.05), **indicates a highly significant difference (*p* < 0.01), and ***indicates an extremely significant difference (*p* < 0.001).

Compared to the time kill curves against the susceptible strain CVCC259 (Figure 1), Tul alone showed reduced efficacy against T32, requiring a longer duration to achieve bacterial clearance. In contrast, MPX displayed comparable activity against both susceptible and resistant strains. The combination therapy further confirmed that MPX significantly potentiates the antibacterial effect of Tul against the resistant strain.

Compared with its activity against the susceptible strain, Tul alone exhibited weaker antibacterial activity against T32, requiring concentrations >2 MIC to prevent regrowth. Conversely, both MPX alone and the combination therapy demonstrated superior efficacy compared to their effects on the susceptible strain. A similar pattern was observed in the kill rates.

## Discussion

Considering the comparatively slow development of antibacterial agents in relation to the rapid emergence of resistant bacterial strains, the enhancement of antibiotic efficacy and the extension of their therapeutic lifespan through the use of antimicrobial synergists represent a more efficient and cost-effective strategy. Accordingly, combination therapy emerges as a viable method to restore the effectiveness of antibiotics that have become less potent. AMPs are small proteins exhibiting a range of biological functions such as membrane disruption, biofilm degradation, and modulation of the host immune response, are identified as promising candidates for next-generation antibiotic adjuvants (Magana et al., 2020; Zharkova et al., 2019). Beyond their intrinsic antimicrobial properties, accumulating evidence suggests that certain AMPs can potentiate the activity of conventional antibiotics. The concurrent use of AMPs and antibiotics not only yields enhanced therapeutic outcomes against drug-resistant bacterial infections but also contributes to reducing the development of further resistance.

Numerous AMPs exert their antimicrobial activity primarily through the disruption of membrane stability, a mechanism that facilitates the enhanced penetration of conventional antibiotics into bacterial cells, thereby promoting synergistic interactions. It has been reported that the AMP P-113 and its derivatives could restore the antibacterial activity of vancomycin against resistant *E. faecalis* and *S. aureus*, neutralize lipopolysaccharides (LPS) secreted by wild-type *E. coli*, and disrupt bacterial cell membranes (Wu et al., 2020). Similarly, Song et al. discovered that the AMP SLAP-S25 could impair membrane integrity, enhancing the antibacterial activity of various AMDs against MDR G^−^ bacteria by up to 1,024-fold, and remained effective against colistin-resistant *K. pneumoniae* harboring the *mcr* gene (Song et al., 2020). Tall et al. found that an ultra-short AMP OW could reduce the MIC of rifampicin against methicillin-resistant *S. aureus* (MRSA) by 85% and that of ampicillin against MDR *Pseudomonas aeruginosa* by 96%, while also degrading biofilms (Tall et al., 2019). Grassi et al. demonstrated that the AMP lin-SB056-1 and its derivative (lin-SB056-1)₂-K could significantly inhibit the formation of *P. aeruginosa* biofilms in combination with EDTA (Grassi et al., 2019). Geitani et al. observed that a cationic AMP combined with AMDs could reduce the MIC of colistin against MRSA and MDR *P. aeruginosa* by 8-fold and that of imipenem by 4-fold, while also reducing the trend of bacterial resistance mutations (Geitani et al., 2019).

APP is a significant porcine respiratory pathogen known for its capacity to form substantial biofilms during growth. Given the critical role that biofilms play in antimicrobial resistance, strategies aimed at inhibiting biofilm formation hold considerable promise for mitigating biofilm-associated resistance (Gondil and Subhadra, 2023). One of the principal mechanisms of the AMP MPX involves the inhibition of APP biofilms (Wang et al., 2020). Moreover, our prior investigations into the MSW of Tul against APP have demonstrated a tendency for resistance development. Accordingly, the present study evaluated the synergistic potential of MPX in combination with Tul against APP, focusing on antibacterial activity, PAE, prevention of resistance mutations, and MSW. The FICI of MPX combined with Tul indicated an additive effect against resistant strains but indifference against susceptible strains. Notably, time-kill assays and kill rate exhibited significant enhancement against both susceptible and resistant strains. This phenomenon may be attributed to resistant mutants increasing biofilm production to sequester Tul, thereby limiting its effective concentration. However, MPX markedly reduces biofilm biomass, which likely contributes to the enhanced antibacterial efficacy of the combination against drug-resistant bacteria. Additionally, AMPs such as MPX are known to inhibit efflux pumps, a principal resistance mechanism, thereby augmenting intracellular antibiotic accumulation and restoring antimicrobial activity (Xia et al., 2021). Consequently, the combined application of MPX and Tul significantly diminished biofilm content in drug-resistant APP strains, increased intracellular Tul concentration, and reduced its efflux, culminating in an additive antibacterial effect.

PAE refers to the phenomenon whereby bacterial growth remains suppressed even after the antimicrobial agent has been eliminated or its concentration has fallen substantially below the MIC. To date, a comprehensive mechanistic explanation for PAE remains elusive. It is hypothesized that PAE may be associated with alterations in bacterial enzyme proteins, metabolic pathways, virulence factors, and adhesion properties following exposure to antimicrobial agents. As a critical pharmacodynamic parameter, PAE plays a pivotal role in guiding the optimization of dosing intervals and minimizing toxic side effects. AMPs hold significant potential for prolonging the PAE of antimicrobial agents. For instance, Ma et al. demonstrated that the self-assembled AMPs FKN and FFN exhibit comparable PAEs against both susceptible and resistant *E. coli* (1.35 to 4.50 h) and *S. aureus* (1.30 to 5.30 h), which is primarily attributed to their enhanced non-specific bacterial binding capacity (Ma et al., 2024). Similarly, Zhang et al. reported that the AMP AP138L-arg26 eradicated *S. aureus* ATCC 43300 within 1.5 h, whereas vancomycin required a minimum of 6 h for clearance. Moreover, the PAEs of AP138L-arg26 (0.9 to 1.91 h) were significantly longer than those of vancomycin (0.27 to 1.18 h) at concentrations ranging from 1 to 4 times the MIC (Zhang et al., 2024). Consistent with these findings, the present study observed that MPX markedly extended the PAE of Tul against APP, increasing it from 0.66 to 1.29 h at 1 MIC and from 1.64 to 2.91 h at 2 MIC. This enhancement may be attributable to the distinct bactericidal mechanisms of the two agents, with their combination resulting in a significant prolongation of the PAE.

Serial passage resistance induction assays are widely employed to evaluate a drug’s potential to select for resistant bacterial strains. Substantial experimental data indicate that AMPs generally exhibit a low propensity to induce resistance. For instance, Chatupheeraphat et al. demonstrated that when *K. pneumoniae* was serially passaged in the presence of 0.5 MIC of ciprofloxacin, colistin, or the AMP K11, the MIC of ciprofloxacin increased by 16-fold after seven passages and by 64-fold after 12 passages (Chatupheeraphat et al., 2023). In contrast, the MIC of AMP K11 fluctuated only slightly over 30 passages, never exceeding a two-fold increase, suggesting a minimal tendency for resistance development against AMP K11. Similarly, Lu et al. reported that *E. coli* cultured under sub-MIC levels of erythromycin exhibited a two-fold increase in MIC by passage 14 and a 16-fold increase by passage 30 (Lu et al., 2022). Conversely, *E. coli* exposed to sub-MIC concentrations of the AMP CATH-1 did not develop resistance over 30 passages. Moreover, when CATH-1 was combined with erythromycin, the MIC increased only two-fold by the 20th passage and remained 16-fold lower at passage 30 compared to erythromycin alone, indicating that CATH-1 can delay the onset of erythromycin resistance. In the current study, resistance induction assays revealed that MPX reduced the MIC of Tul against APP to 0.5 MIC after 20 passages, and the MIC in the combination treatment group remained at 0.5 MIC for the first 22 passages. These results suggest that MPX possesses a certain capacity to suppress the emergence of resistant bacterial strains.

The MSW theory plays a pivotal role in the rational design of dosing regimens aimed at preventing antimicrobial resistance and has been the subject of extensive investigation (Lozano-Huntelman et al., 2020; Shi et al., 2019). Nonetheless, the MSW framework exhibits certain limitations. For instance, bacterial populations at high concentrations (>10^9^ CFU/mL) can produce biofilms that interfere with the accurate determination of the MPC. Additionally, an excessively broad MSW increases the likelihood that drug concentrations will fall within this window, thereby facilitating the emergence of resistance mutations. Conversely, an excessively elevated MPC may result in overdosing, which poses risks of toxicity or mortality in animal models. Consequently, strategies aimed at narrowing or closing the MSW are critical for mitigating the occurrence of single-step bacterial resistance mutations. One promising approach involves the combination of AMPs with conventional antibacterial agents to effectively close the MSW. Roson-Calero et al. (2025) investigated the antibacterial efficacy of a novel AMP in combination with tetracycline antibiotics against *P. aeruginosa*. Their findings demonstrated a significant reduction in the MIC of minocycline against wild-type *P. aeruginosa* PAO1, decreasing from 4–8 to 0.5–1.0 μg/mL when co-administered with 100 μg/mL of AMP RW01. Moreover, the MPC of the combination therapy was markedly lowered, from 32 to 64 μg/mL with minocycline alone to 8 μg/mL with RW01 co-treatment. Complementary research by Ma et al. revealed that self-assembled AMPs exhibited comparable MPC values against both susceptible and resistant strains of *E. coli* and *S. aureus*, with no significant differences observed in the SI between standard laboratory strains and clinical isolates (Ma et al., 2024). Specifically, the SI values of AMPs FKN and FFN against *E. coli* and *S. aureus* ranged from 2.0 to 4.8 and 2.4 to 4, respectively. In this investigation, the combined administration of the two drugs resulted in a reduction of SI for Tul from 64 to 32, and for MPX from 32 to 4, demonstrating that MPX possesses a potential capacity to inhibit resistance mutations. Furthermore, the FICI calculated based on the MPC was markedly lower (0.375) compared to the FICI derived from the MIC-based combination index (1.5). A lower FICI value indicates a greater likelihood that multiple sensitive targets of a compound are in close proximity, thereby enhancing the compound’s effectiveness in preventing resistance development, consistent with the MSW and MPC theoretical frameworks (Xu et al., 2018). Consequently, these findings suggest that MPX exhibits a potential advantage in mitigating the emergence of resistance mutations.

In summary, this study found that MPX can enhance the antibacterial activity and kill rate of Tul against both susceptible and resistant APP. The combination also can prolong the PAE of Tul against APP. Moreover, MPX demonstrated the ability to inhibit the emergence of resistance mutations in susceptible bacterial populations at sub-inhibitory concentrations and to reduce the MSW of Tul. However, this study is subject to certain limitations, including its exclusive reliance on *in vitro* experiments, absence of pharmacokinetic/pharmacodynamic (PK/PD) integration, lack of animal model validation, and omission of biofilm assays. Furthermore, the mechanistic explanations provided remain theoretical or speculative and are not directly supported by empirical data from this investigation. Consequently, future research incorporating PK/PD modeling, biofilm assessments, resistance gene sequencing following bacterial passage, and mechanistic studies—such as evaluations of membrane permeability and efflux pump inhibition—will be necessary to elucidate the underlying mechanisms of the MPX and Tul combination against APP.

## Data Availability

The raw data supporting the conclusions of this article will be made available by the authors, without undue reservation.
